# Transactions of Medical Association of Alabama, 1875

**Published:** 1876-05

**Authors:** 


*Transactions of Medical Association of Alabama*, 1875—*Pages* 260.—This is a large and creditable Report from the State of Alabama. Few State organizations, indeed, have done so well. Many able and interesting papers are reporte l, among which we will mention as of special interest, the President's Address, by Dr. Demont; Dr. Ketchum's *Oration on Ethics;* Dr. Bizzell on *Climate of the U. S. with reference to Consumption and Pneumonia;* Dr. Riggs on *Malaria;* Dr. Peterson on *Gynæcology;* Dr. Cochran on *Small Pox Epidemic in Mobile, etc.*

